# Comparative Analysis of *Streptococcus pneumoniae* Type I Restriction-Modification Loci: Variation in *hsdS* Gene Target Recognition Domains

**DOI:** 10.3390/pathogens9090712

**Published:** 2020-08-29

**Authors:** Melissa B. Oliver, W. Edward Swords

**Affiliations:** 1 Department of Medicine, Division of Pulmonary, Allergy, and Critical Care Medicine University of Alabama at Birmingham, Birmingham, 35294 AL, USA; mboliver@uabmc.edu; 2 Gregory Fleming James Cystic Fibrosis Research Center, University of Alabama at Birmingham, Birmingham, 35294 AL, USA

**Keywords:** *Streptococcus pneumoniae*, pneumococcus, phase variation, *hsdS*

## Abstract

*Streptococcus pneumoniae* (pneumococcus) is a respiratory commensal pathogen that causes a range of infections, particularly in young children and the elderly. Pneumococci undergo spontaneous phase variation in colony opacity phenotype, in which DNA rearrangements within the Type I restriction-modification (R-M) system specificity gene *hsdS* can potentially generate up to six different *hsdS* alleles with differential DNA methylation activity, resulting in changes in gene expression. To gain a broader perspective of this system, we performed bioinformatic analyses of Type I R-M loci from 18 published pneumococcal genomes, and one R-M locus sequenced for this study, to compare genetic content, organization, and homology. All 19 loci encoded the genes *hsdR, hsdM, hsdS,* and at least one *hsdS* pseudogene, but differed in gene order, gene orientation, and *hsdS* target recognition domain (TRD) content. We determined the coding sequences of 87 *hsdS* TRDs and excluded seven from further analysis due to the presence of premature stop codons. Comparative analyses revealed that the TRD 1.1, 1.2, and 2.1 protein sequences had single amino acid substitutions, and TRD 2.2 and 2.3 each had seven differences. The results of this study indicate that variability exists among the gene content and arrangements within Type I R-M loci may provide an additional level of divergence between pneumococcal strains, such that phase variation-mediated control of virulence factors may vary significantly between individual strains. These findings are consistent with presently available transcript profile data.

## 1. Importance

Phase variation is common among bacterial pathogens and usually involves a “switch” between different subpopulations. For example, pneumococcal populations undergo phase variation via recombination events within the Type I restriction-modification locus yielding alternate alleles of the target specificity subunit *hsdS*, resulting in subpopulations with differential DNA methylation and gene expression. Here we present results of bioinformatic analyses to profile and compare the Type I R/M loci from a panel of diverse pneumococcal strains. The potential implication of HsdS genetic variation in the rate and targets of methylase-mediated phase variation could include specific gene silencing, and/or altered gene expression. Sequence variation in *hsdS* genes encoding target recognition subunits, as well as variation in the number of flanking *hsdS’* pseudogenes within the relevant genomic locus, suggests additional levels of diversity between strains. This interpretation is consistent with the observed variability between strains in terms of transcript profiles and rates of phase variation.

## 2. Introduction

*Streptococcus pneumoniae* (pneumococcus) is a significant opportunistic pathogen that can cause a variety of localized infections of the respiratory mucosa, as well as serious invasive diseases such as sepsis and meningitis [1,2,3]. Invasive pneumococcal infections are associated with a high degree of morbidity and mortality, despite the availability of vaccines and antibiotics [4,5]. Pneumococcal carriage in the nasopharynx and upper airways is quite common, especially among children, and represents the reservoir and initial stage for infection [6,7,8,9,10,11,12]. The ability of pneumococci to rapidly adapt to different host environments is important to combat host immune defenses. Phase variation between differentiated phenotypic states is a common mechanism by which pathogenic bacteria can adapt rapidly to changing host environments. Pneumococcal phase variation is estimated to occur at a rate of 10^−3^ to 10^−6^ per generation (markedly greater than the 10^−8^ per generation rate for spontaneous mutation) and is visible as opaque or transparent colony phenotypes when viewed under oblique light [13]. Opaque variants are typically recovered from invasive infection sites and have increased virulence-associated phenotypes such as resistance to complement and phagocytic killing, whereas transparent variants are associated with asymptomatic colonization and localized disease [13,14,15,16]. Clinical pneumococcal isolates contain a heterogeneous mixture of both colony phenotypes, but since the phenotypes are not genetically “locked,” and thus they can freely switch back and forth, resulting in a variable and constantly changing proportion of each phenotype within the overall bacterial population. Pneumococcal phase variation is a complex process that has been the subject of intense interest and study over a number of years [13,14,17,18,19,20,21].

The genetic mechanism for pneumococcal phase variation is based on recombination-mediated diversity between six different alleles of the hsd Type I restriction-modification (R-M) locus [22,23,24]. This locus encodes the target specificity gene *hsdS*, methyltransferase gene *hsdM*, and restriction gene *hsdR* that encode the HsdS, HsdM, and HsdR subunits. The subunits assemble into a heteromeric enzyme complex that functions to specifically methylate the bacterial genomic DNA and destroy foreign DNA [25]. The HsdS subunit has two different DNA target recognition domains (designated as TRD 1 and TRD 2) which direct recognition of specific sequence motifs that are then methylated by the HsdM subunit [22,23]. The number of recognition sequence repeats in the genome determines the DNA methylation pattern, which in turn influences gene expression. This mechanism is further complicated by the presence of *hsdS* pseudogenes with divergent TRD sequences in the R-M locus. To date, two versions of TRD 1 (named 1.1 and 1.2) and three versions of TRD 2 (named 2.1, 2.2, and 2.3) have been characterized. Recombination events between these TRD sequences can produce six predicted *hsdS* genes, resulting in six unique HsdS subunits that each have unique bacterial DNA methylation patterns [22,23].

HsdS-mediated DNA methylation was shown to change gene expression linked to phase variation of colony morphology [22,23,24]. Notably, we found that specific TRD alleles conferred different phenotypes in different pneumococcal strain backgrounds. One explanation for this phenotypic difference was that genetic variation may have existed in the *hsdS* TRD coding sequences. Thus, in this study, we performed a bioinformatics analysis to compare the DNA sequences of TRD 1.1, 1.2, 2.1, 2.2, and 2.3 between 19 pneumococcal genetic backgrounds and found that *hsdS* TRDs were greater than 96% similar at the protein level. This led us to hypothesize that the differences in TRD sequences could affect target sequence specificity and/or activity mediating DNA methylation. Notably, the published transcript profiles between phase-locked strains indicate significant variation in *hsdS* phase-types in different pneumococcal strain backgrounds; i.e., specific *hsdS* alleles did not uniformly result in the same gene expression profile. In order to understand how methylase-mediated phase variation may have differential effects in different strains, we performed a comprehensive bioinformatic comparison of the relevant genes from a wide array of pneumococcal genomic sequences. The results indicate that while there is a high degree of sequence conservation among *hsd* genes, there were key divergences in gene arrangement within the loci. Potential implications for these differences in rearrangements in control of phase variation are discussed.

## 3. Results

### 3.1. S. pneumoniae hsd Type I Restriction-Modification (R-M) Loci Are Genetically Diverse

To gain a broad perspective of the Type I R-M locus in pneumococci, we first aimed to acquire a diverse genetic dataset. We performed a nucleotide BLAST search on the NCBI GenBank Database using the *S. pneumoniae* TIGR4 *hsdS* gene (1569 bp) as the query sequence. The search summary showed BLAST hits on 73 subject sequences. Two partial matching subject sequences belonged to *Fusobacterium nucleatum* but they were excluded because no Type I R-M system was detected in either *F*. *nucleatum* genome. Of the remaining 71 subject sequences, we identified 38 candidate data sets that had complete genome data available. Each candidate data set was carefully screened until we collected a representative pool of 18 complete genomes that differed in serotype, body site of origin, and country of origin (Table 1). No information was available regarding the ability of the strains to undergo phase variation of colony phenotype. 

**Figure 1:**
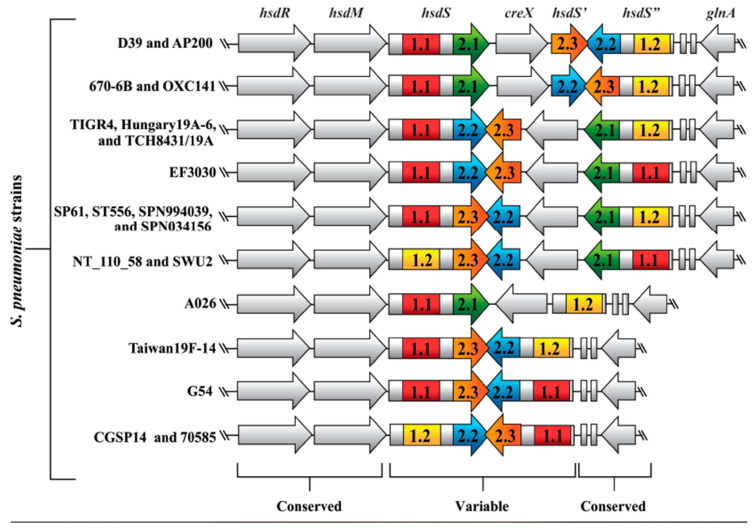
Homology comparison of the Type I restriction-modification locus from 19 different pneumococcal strains. GenBank accession numbers are listed in Table 1. Strain names are shown on the left. The *hsdS* target recognition domains 1.1 (red), 1.2 (yellow), 2.1 (green), 2.2 (blue), and 2.3 (orange) are shown. Strains EF3030 and G54 encoded two identical copies of TRD 1.1, so the second copy in *hsdS*’’ was labeled TRD 1.1′. The two small coding sequences between *hsdS*’’ and *glnA* are hypothetical genes.

We first aimed to determine if pneumococci contained multiple copies of the Type I R-M locus. To address this, each genome was carefully examined and found to contain a single copy of the locus. To determine the genetic content, organization and homology between the R-M loci, the coding sequences in each genome were downloaded from the GenBank database and carefully annotated using a DNA editing program. An annotated genomic sequence for *S. pneumoniae* EF3030 was not available, so its R-M locus was sequenced for this study. We found that all 19 R-M loci encoded the restriction gene *hsdR,* methylase gene *hsdM,* specificity gene *hsdS,* and at least one *hsdS* pseudogene, and ranged in size from 7.2 to 8.5 kb. Since we knew from our previous study that *S. pneumoniae* D39 and *S. pneumoniae* TIGR4 *hsdS* target recognition domains (TRD) were distributed differently, we wondered whether this was true in other pneumococcal strains. To address this, the 19 R-M loci were examined and found to encode a total of 87 *hsdS* TRDs that differed in location. With this information in hand, we were able to create schematic maps for each strain and easily assess shared features (Figure 1).

Comparative analyses revealed that the R-M loci differed in gene order, gene orientation, and *hsdS* TRD content. Some strains (e.g., *S. pneumoniae* D39 and *S. pneumoniae* AP200) had identical genetic content and were grouped together resulting in a total of 10 unique locus “types.” Most strains (13 out of 19) encoded all five TRDs (1.1, 1.2, 2.1, 2.2, and 2.3). Two strains encoded two identical copies of TRD 1.1, but completely lacked TRD 1.2, while four strains encoded only some TRDs. Four out of 19 strains lacked the recombinase gene *creX* and TRD 2.1 suggesting that the two genetic factors are commonly linked and are dispensable in this system. Overall, we concluded that the Type I R-M locus was a conserved feature in pneumococci that was susceptible to high rates of recombination-mediated mutation in the *hsdS* gene and pseudogenes. Moreover, due to the variable number of *hsdS* TRD pseudogenes, the potential exists for variation in numbers of potential allelic combinations between strains.

### 3.2. S. pneumoniae hsdS Coding Sequences Were Highly Homologous

Since we and others showed that the combination of *hsdS* TRDs within a single strain directly affected pneumococcal phase variation, we next aimed to determine whether genetic variation existed within the *hsdS* TRD coding sequences. To address this, all 87 *hsdS* TRD protein-coding sequences were determined. Seven sequences had premature stop codons and were excluded from further analysis. We next tallied the total number of in-frame sequences to compare for each TRD: TRD 1.1 (*n* = 18), TRD 1.2 (*n* = 15), TRD 2.1 (*n* = 15), TRD 2.2 (*n* = 17), and TRD 2.3 (*n* = 15). A summary is listed in Table 2.

Comparative analyses using a protein alignment program revealed a high level of similarity (>96%) between sequences belonging to the same TRD. In the 18 TRD 1.1 comparisons, a single amino acid substitution was detected at position one in strain G54 (Phe1Ile) resulting in an overall 99.4% similarity. In the 15 TRD 1.2 comparisons, a single amino acid substitution was identified in *S. pneumoniae* SPN994039 and *S. pneumoniae* OXC141 (Ala134Pro) resulting in a 99.2% similarity. In the 15 TRD 2.1 comparisons, three unique mutations were present at position 101: eight strains encoded 101-Gly, six strains encoded 101-Ala, and one strain encoded 101-Val resulting in 99.4% similarity. In the 17 TRD 2.2 comparisons and 15 TRD 2.3 comparisons, seven different mutations sites resulted in a 96.7% similarity for each data set. 

**Figure 2:**
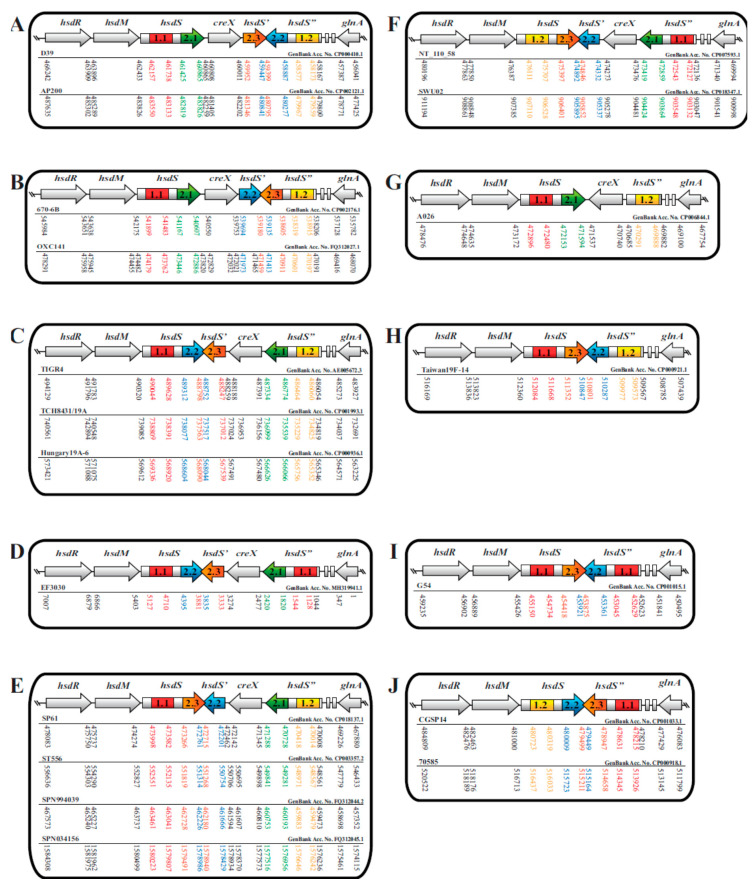
Schematic showing DNA coding sequences for all genes in 18 Type I R-M loci. This figure is a detailed extension of Figure 1. Below each locus, the pneumococcal strain name, its GenBank Acc. No., and DNA open reading frame positions are shown. DNA positions listed in black font were reported by the GenBank database. DNA positions shown in grey or color were calculated for this study. To aid in visualization, the TRD coding sequence positions were color-matched to the open reading frame schematic.

**Figure 3:**
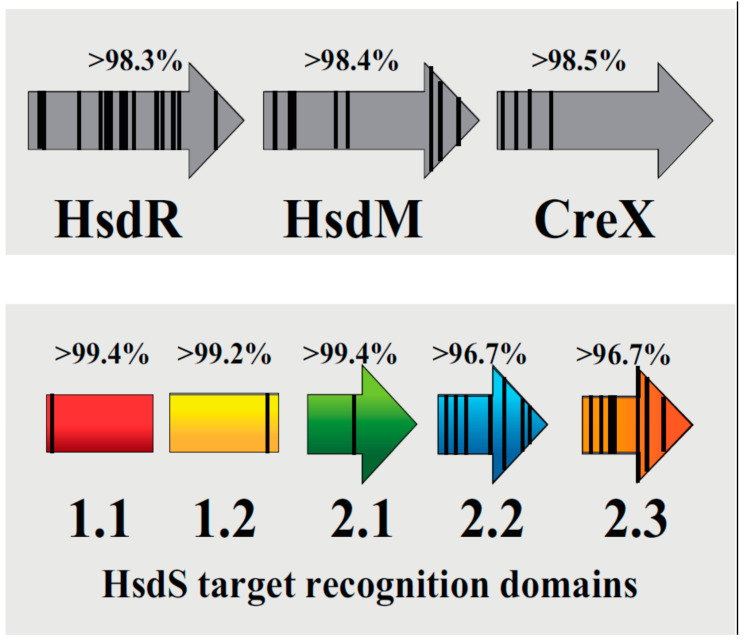
Schematic showing similarity between coding sequences in HsdR, HsdM, CreX, and HsdS target recognition domains. The protein-coding sequences for all HsdR, HsdM, CreX, and TRDs in the 19 pneumococcal strains were carefully compared and analyzed. Vertical lines indicate that at least one amino acid mutation was detected at that position. The total number of matching residues were used to determine % similarity (shown above each coding sequence).

We next wondered whether other genes in the Type I R-M locus were different from one another (Figure 2, Table 3). To address this, the protein-coding sequences for the restriction subunit HsdR, the methylase subunit HsdM, and the recombinase unit CreX were determined and compared sequence alignments of 18 HsdR, 18 HsdM, and 15 CreX protein sequences revealed a high level of similarity (>98%) between the subunits (Table 2). Based on the data from these analyses, we were able to create a summary schematic that mapped mutation positions and the overall percent similarity (Figure 3). It became apparent that the *hsdS* genes and *hsdS* pseudogenes had more differences in the TRD 2 coding sequences. It is possible the variation observed in TRD 2 may indicate a driving role in determining DNA methylation specificity as compared to TRD 1. The possibility also exists that variation in the *hsdR* and *hsdM* genes could affect inter-subunit interactions to affect the fidelity of DNA methylation. 

## 4. Discussion

*S. pneumoniae* phase variation of colony morphology is mediated by site-specific recombination of the *hsdS* gene in a Type I restriction-modification (R-M) system, which alters DNA methylation and ultimately results in differential gene expression [22]. Although the combination of *hsdS* target recognition domains (TRD) were shown to be associated with phase variation in different strains [22,23,24], we aimed to investigate whether the TRD DNA and protein-coding sequences were conserved in different strains. To address this, we conducted a comparative analysis study of 19 *S. pneumoniae* Type I R-M loci and determined their *hsdS* TRD content, organization, and sequence identity. 

The identification of a Type I R-M system in 19 diverse *S. pneumoniae* genetic backgrounds was significant because it suggested that this system may act as a conserved and underappreciated virulence factor. Within this dataset, we characterized ten unique R-M locus “types” that differed in *hsdS* genetic content. Six R-M locus “types” were expressed by more than two strains each, indicating that some R-M loci may be more favorable than others and are maintained in a population. Since all ten “types” encoded at least three TRDs each, we concluded that the preservation of many TRDs could increase the likelihood of generating an advantageous recombinant *hsdS* variant with a better chance of survival. However, the four strains lacking TRD 2.1 and creX may have reduced potential for the generation of *hsdS* allele variants.

The high amount of *hsdS* genetic diversity found in the 19 Type I R-M loci led us to investigate the sequence identity of 87 different *hsdS* TRD protein sequences. We demonstrated that they were very similar, but not always identical. For example, the protein sequences of TRD 1.1, 1.2, and 2.1 had single amino acid differences while those of TRD 2.2 and 2.3 had seven differences each. These findings are important because they suggest that phase variation-specific epigenetic regulation via DNA methylation may be mediated by small genetic differences, particularly in the second TRD. One potential implication is that a mutation in an *hsdS* TRD could alter the HsdS recognition sequence, result in differential DNA methylation, and ultimately alter gene expression. This could lead to changes in bacterial fitness due to increased or decreased virulence factor expression or gene silencing. It is also possible that sequence differences in the HsdR, HsdM, and HsdS TRDs can mediate how the subunits work together as a multimeric complex. Future studies would be necessary to determine how the subunits fit together and whether the mutation sites map to the predicted multimeric interface.

*S. pneumoniae* phase variation occurs every 10^−3^ to 10^−6^ per generation and typically results in opaque or transparent colony phenotypes [13]. It remains unclear whether certain *hsdS* types, or mutations in *hsdS* TRDs, can alter the rate of phase switching in pneumococci. We propose that it would in a strain-specific manner and would be highly dependent on the type and number of surface-expressed factors encoded. It is difficult to speculate on potential rates of phase switching when two studies have shown that pneumococcal strains encoding a single *hsdS* allele produced monomorphic colonies that were either all opaque or all transparent [23,24]. Spontaneous phenotypic variation is a common theme in pathogenic bacteria to increase biological fitness under changing conditions. Bacterial adaptation occurs by either alterations in DNA sequences (genetic mutations), or differences in DNA methylation (epigenetic regulation). Intra-host bacterial evolution is primarily driven by spontaneous mutations (e.g., slipped-strand mispairing, recombination events, and point mutations) in surface-expressed factors/antigens [41]. For example, a random on/off slipped-strand mispairing over simple sequence repeats in genes encoding phosphorylcholine and other lipooligosaccharide antigens in *Haemophilus influenzae* can result in high-frequency phase variation (10^−2^/cell per generation) [42,43,44,45]. This mechanism has also been reported in *Helicobactor pylori* [46], *Escherichia coli* [47], and *Staphylococcus aureus* [48,49]. Recombination-mediated mutations resulting in phase variants have been reported for *Salmonella* [50], *Mycoplasma pneumoniae* [51,52], and *N. meningitidis* [53,54]. *Neisseria gonorrhoeae* undergoes low-frequency phase variation (10^−6^ /cell per generation) [55]. Small colony variants of *Pseudomonas aeruginosa* typically arise due to spontaneous mutations [56]. These examples highlight how simple genetic modifications in a variety of human pathogens can alter their fitness. 

The major limitation of this study was the relatively small number of complete pneumococcal genomes candidates analyzed (*n* = 18). Another limitation was the query sequence used to identify candidate genome data. In this analysis, we clearly showed that some pneumococcal R-M loci lack one or more *hsdS* TRDs. By searching only for sequences that encoded *S. pneumoniae* TIGR4 *hsdS*, which only encoded TRDs 1.1 and 2.2, we may have excluded some pneumococcal strains. A separate more inclusive search of all available genomes that encode at least one TRD would allow us to gain a comprehensive understanding of genetic variation in the R-M locus. By comparing the DNA methylation patterns and transcript profile, one could potentially develop a more refined understanding of phase variation-specific expression patterns. Alternatively, this system could be used to aid in the alteration of virulence factor expression or gene silencing which could help refine the expression of factors involved in host-pathogen interactions. Overall, our findings may help explain how pathogenic pneumococci can rapidly undergo intra-host adaptations in order to survive in diverse and rapidly changing environments. Understanding the relationship between the *hsdS* TRD combination, sequence identity, and specific recognition sequence may aid in a better understanding of how gene expression is altered. The findings in this study may serve as a model for future studies of host-pathogen interactions.

## 5. Materials and Methods

### 5.1. Sequence Analysis

Type I restriction-modification (R-M) system sequence data from 18 *S. pneumoniae* strains were acquired from the GenBank database. The sequencing of the *S. pneumoniae* EF3030 (R-M) system was performed at the UAB Heflin Center for Genomic Science. The 19 Type I R-M systems were carefully annotated using the DNA alignment program A Plasmid Editor V2.0.47. Each *hsdS* target recognition domain coding sequence was translated to protein sequence using A Plasmid Editor. Multiple protein sequence alignments were performed using EBI Clustal Omega, Cambridge, UK (1.2.4; http://www.clustal.org/omega/). The exclusion criteria for data analysis was the presence of a premature stop codon. The list of excluded samples included *S. pneumoniae* strains: AP200 (TRD 1.2 and 2.2), G54 (TRD 2.3), SWU02 (TRD 1.2 and 2.3), OXC141 (TRD 1.1), and EF3030 (TRD 2.3). Strains G54 and EF3030 each had two identical copies of TRD 1.1, so only one copy of the sequence was used in this analysis. 

### 5.2. Accession Numbers

Listed here are *S. pneumoniae* strain names and their GenBank Accession numbers in parentheses: *S. pneumoniae* D39 (CP000410.1), *S. pneumoniae* 70585 (CP000918.1), *S. pneumoniae* TIGR4 (AE005672.3), *S. pneumoniae* 670-6B (CP002176.1), *S. pneumoniae* AP200 (CP002121.1), *S. pneumoniae* CGSP14 (CP001033.1), *S. pneumoniae* TCH8431/19A (CP001993), *S. pneumoniae* Hungary19A-6 (CP000936.1), *S. pneumoniae* SP61 (CP018137.1), *S. pneumoniae* ST556 (CP003357.2), *S. pneumoniae* Taiwan19F-14 (CP000921.1), *S. pneumoniae* G54 (CP001015.1), *S. pneumoniae* SWU02 (CP018347.1), *S. pneumoniae* A026 (CP006844.1), *S. pneumoniae* OXC141 (FQ312027.1), *S. pneumoniae* SPN034156 (FQ312045.1), *S. pneumoniae* SPN994039 (FQ312044.2), *S. pneumoniae* EF3030 (MH319941.1), and *S. pneumoniae* NT_110_58 (CP007593.1).

## Figures and Tables

**Table 1:** Descriptive table of 19 *S. pneumoniae* genomes analyzed for this study. The strains differed in serotype, body site origin, and country of origin. GenBank Accession numbers are listed. ND, not determined.

Strain	GenBank No.	Serotype	Body Site Origin	Country of Origin	Reference
NT_110_58	CP007593.1	NT	Nasopharynx	Switzerland	[26]
D39	CP000410.1	2	Blood	ND	[27]
OXC141	FQ312027.1	3	Carriage	UK	[28]
SPN034156	FQ312045.1	3	ND	Italy	[28]
SPN994039	FQ312044.2	3	ND	ND	[28]
70585	CP000918.1	4	ND	ND	[29]
TIGR4	AE005672.3	4	Blood	Norway	[30]
670-6B	CP002176.1	6B	ND	Spain	[31]
AP200	CP002121.1	11A	Meningitis	Italy	[32]
CGSP14	CP001033.1	14	ND	China	[33]
TCH8431/19A	CP001993.1	19A	ND	USA	[34]
Hungary19A-6	CP000936.1	19A	ND	Hungary	[35]
SP61	CP018137.1	19A	Thorax	Germany	[36]
ST556	CP003357.2	19F	Otitis media	USA	[37]
Taiwan19F-14	CP000921.1	19F	ND	Taiwan	[28]
G54	CP001015.1	19F	Respiratory	Italy	[38]
SWU02	CP018347.1	19F	Sputum	China	[39]
A026	CP006844.1	19F	ND	China	[40]
EF3030	MH319941.1	19F	Otitis media	USA	This study

**Table 2:** Genetic content and similarity comparison of 19 different Type I R-M loci. The loci were analyzed for the presence of the restriction gene *hsdR*, the methylase gene *hsdM*, the recombinase gene *creX*, and the five specificity-gene *hsdS* TRDs. The plus and minus symbols indicate the coding sequence was either present or not detected, respectively.

				*hsdS* Target Recognition Domain				
*S. pneumoniae* Strain	*hsdR*	*hsdM*	*creX*	1.1	1.2	2.1	2.2	2.3
D39	+	+	+	+	+	+	+	+
TIGR4	+	+	+	+	+	+	+	+
TCH8431/19A	+	+	+	+	+	+	+	+
Hungary19A-6	+	+	+	+	+	+	+	+
SP61	+	+	+	+	+	+	+	+
ST556	+	+	+	+	+	+	+	+
SPN034156	+	+	+	+	+	+	+	+
SPN994039	+	+	+	+	+	+	+	+
NT_110_58	+	+	+	+	+	+	+	+
670-6B	+	+	+	+	+	+	+	+
OXC141	+	+	+	+ ^a^	+	+	+	+
AP200	+	+	+	+	+ ^a^	+	+ ^a^	+
SWU02	+	+	+	+	+ ^a^	+	+	+ ^a^
70585	+	+	+	+	+	-	+	+
SP14	+	+	-	+	+	-	+	+
Taiwan19F-14	+	+	-	+	+	-	+	+
A026	+	+	-	+	+	+	-	-
G54	+	+	-	+ ^b^	-	-	+	+ ^a^
EF3030	+	+	+	+ ^b^	-	+	+	+ ^a^
Sum	19	19	15	19	17	15	18	18
Included for analysis	18	18	15	18	15	15	17	15
Length (amino acid)	777	487	265	179	135	186	186	183
Matching residues	763	479	261	178	134	185	180	177
Similarity (%)	98.3	98.4	98.5	99.4	99.2	99.4	96.7	96.7

^a^, sequence contained a premature stop codon and was excluded from further analysis; ^b^, sequence was found in two separate locations in the locus; both copies were 100% identical.

**Table 3:** *S. pneumoniae* Type I restriction-modification system genetic location. The DNA coding sequences for each gene are listed for convenience.

*S. pneumoniae* Strain GenBank	D39 CP000410.1		AP200 CP002121.1		670-6B CP002176.1		OXC141 FQ312027.1		TIGR4 AE005672.3		Hungary19A-6 CP000936.1		TCH8431/19A CP001993.1		SP61 CP018137.1		ST556 CP003357.2	
	Start	End	Start	End	Start	End	Start	End	Start	End	Start	End	Start	End	Start	End	Start	End
**Locus**	456,041	466,242	477,425	487,635	535,782	545,984	468,070	478,291	483,927	494,129	563,225	573,421	732,691	742,894	467,880	478,083	546,433	556,636
*hsdR*	466,242	463,909	487,635	485,302	545,984	543,651	478,291	475,958	494,129	491,796	573,421	571,088	742,894	740,561	478,083	475,750	556,636	554,303
*hsdM*	463,896	462,433	485,289	483,826	543,638	542,175	475,945	474,455	491,783	490,320	571,075	569,612	740,548	739,085	475,737	474,274	554,290	552,827
*hsdS*	462,433	460,865	483,826	482,259	542,175	540,607	474,482	473,820	490,320	488,752	569,612	568,044	739,085	737,517	474,274	472,709	552,827	551,262
*creX*	460,808	460,011	482,202	481,405	540,550	539,753	472,032	472,829	487,391	488,188	566,683	567,480	736,156	736,953	471,345	472,142	549,898	550,695
*hsdS’*	460,000	459,401	481,295	480,789	539,742	539,134	471,413	472,021	488,259	488,798	567,491	568,096	737,024	737,569	472,462	472,761	550,706	551,314
*hsdS’’*	458,167	459,447	479,800	480,693	538,206	539,180	470,191	471,465	486,054	487,334	565,346	566,626	734,819	736,099	470,008	471,288	548,561	549,841
**Hypothetical**	457,860	457,997	479,245	479,382	537,224	537,343	469,883	470,020	485,746	485,883	565,038	565,175	734,366	734,217	469,322	469,441	548,253	548,390
**Hypothetical**	457,483	457,602	478,951	479,100	537,601	537,738	469,506	469,625	485,488	485,369	564,780	564,661	734,133	734,252	469,406	469,555	547,959	548,108
*glnA*	456,041	457,387	477,425	478,771	535,782	537,128	468,070	469,416	483,927	485,273	563,225	564,571	732,691	734,037	467,880	469,226	546,433	547,779
**TRD 1.1**	461,738	462,157	483,133	483,550	541,483	541,899	473,762	474,179	489,628	490,044	568,920	569,336	738,391	738,809	473,582	473,998	552,135	552,551
**TRD 1.2**	458,173	458,577	479,559	479,967	537,915	538,319	470,197	470,601	486,060	486,464	565,352	565,756	734,825	735,229	470,014	470,418	548,567	548,971
**TRD 2.1**	460,865	461,425	482,259	482,819	540,607	541,167	472,886	473,446	486,774	487,334	566,066	566,626	735,539	736,099	470,728	471,288	549,281	549,841
**TRD 2.2**	458,887	459,447	480,277	480,841	539,134	539,694	471,413	471,973	488,752	489,312	568,044	568,604	737,517	738,077	472,201	472,761	550,754	551,314
**TRD 2.3**	459,399	459,952	480,795	481,346	538,605	539,180	470,911	471,459	488,247	488,798	567,539	568,090	737,012	737,563	472,715	473,266	551,268	551,819
***S. pneumoniae* Strain**	**n SPN994039**		**SPN034156**		**NT_110_58**		**SWU02 ^a^**		**A026**		**Taiwan19F-14**		**G54**		**CGSP14**		**70585**	
**GenBank**	**FQ312044.2**		**FQ312**	**045.1**	**CP007593.1**		**CP018347.1**		**CP006844.1**		**CP000921.1**		**CP001015.1**		**CP001033.1**		**CP000918.1**	
	**start**	**End**	**start**	**End**	**start**	**End**	**start**	**End**	**start**	**End**	**start**	**End**	**start**	**End**	**start**	**End**	**start**	**End**
**Locus**	457,352	467,573	1,574,115	1,584,308	469,994	480,196	900,998	911,194	467,754	476,981	507,439	516,169	450,495	459,235	476,083	484,809	511,799	520,522
*hsdR*	467,573	465,240	1,584,308	1,581,975	480,196	477,863	911,194	908,861	476,981	474,648	516,169	513,836	459,235	456,902	484,809	482,476	520,522	518,189
*hsdM*	465,227	463,737	1,581,962	1,580,499	477,850	476,387	908,848	907,385	474,635	473,172	513,823	512,360	456,889	455,426	482,463	481,000	518,176	516,713
*hsdS*	463,764	462,174	1,580,499	1,578,934	474,840	476,387	907,385	905,852	473,172	471,594	512,360	510,795	455,426	454,332	481,000	479,450	516,713	515,163
*creX*	460,810	461,607	1,577,573	1,578,370	473,476	474,273	904,481	905,278	471,537	470,740	Absent	Absent	Absent	Absent	Absent	Absent	Absent	Absent
*hsdS’*	461,594	462226	1,578,934	1,578,986	Absent	Absent	905,337	905,895	Absent	Absent	Absent	Absent	Absent	Absent	Absent	Absent	Absent	Absent
*hsdS’’*	459,473	460,753	1,576,236	1,577,516	472,136	473,419	903,047	904,424	469,882	470,685	509,567	510,847	452,623	453,921	478,210	479,502	513,926	514,345
**Hypothetical**	459,165	459,302	1,575,928	1,576,065	471,813	471,950	902,014	902,151	469,574	469,711	508,965	509,114	451,937	452,056	477,902	478,039	513,319	513,468
**Hypothetical**	458,788	458,907	1,575,551	1,575,670	471,520	471,669	901,637	901,756	469,220	469,429	508,881	509,000	452,021	452,170	477,524	477,643	513,235	513,354
*glnA*	457,352	458,698	1,574,115	1,575,461	469,994	471,340	900,998	901,541	467,754	469,100	507,439	508,785	450,495	451,841	476,083	477,429	511,799	513,145
**TRD 1.1**	463,041	463,461	1,579,807	1,580,223	472,127	472,543	903,132	903,548	472,480	472,896	511,668	512,084	454,734	455,150	478,215	478,631	513,926	514,345
**TRD 1.1’**	Absent	Absent	Absent	Absent	Absent	Absent	Absent	Absent	Absent	Absent	Absent	Absent	452,629	453,045	Absent	Absent	Absent	Absent
**TRD 1.2**	459,479	459,883	1,576,242	1,576,646	475,707	476,111	906,528	907,110	469,888	470,291	509,573	509,977	Absent	Absent	480,319	480,723	516,033	516,437
**TRD 2.1**	460,193	460,753	1,576,956	1,577,516	472,859	473,419	903,864	904,424	471,594	472,153	Absent	Absent	Absent	Absent	Absent	Absent	Absent	Absent
**TRD 2.2**	461,666	462,226	1,578,429	1,578,986	474,332	474,892	905,337	905,895	Absent	Absent	510,287	510,847	453,361	453,921	479,449	480,009	515,164	515,723
**TRD 2.3**	462,180	462,728	1,578,940	1,579,491	474,846	475,397	905,852	906,401	Absent	Absent	510,801	511,352	453,875	454,418	478,947	479,499	514,658	515,211

^a^, Italicized data indicate coding sequences determined for this study; ^b^, the DNA data for strain SWU2 (GenBank Acc. No. CP018347.1) was not annotated, so its locus coding sequences were determined for this study. ^c^, absent means the indicated coding sequences were not detected in the dataset.
